# Antimicrobial and Functional Properties of Duckweed (*Wolffia globosa*) Protein and Peptide Extracts Prepared by Ultrasound-Assisted Extraction

**DOI:** 10.3390/foods11152348

**Published:** 2022-08-05

**Authors:** Natchaya Duangjarus, Weerachai Chaiworapuek, Chitsiri Rachtanapun, Pitiporn Ritthiruangdej, Suvimol Charoensiddhi

**Affiliations:** 1 Department of Food Science and Technology, Faculty of Agro-Industry, Kasetsart University, Bangkok 10900, Thailand; 2 Advance Technology for Food Safety Specialty Unit, Kasetsart University, Bangkok 10900, Thailand; 3 Department of Mechanical Engineering, Faculty of Engineering, Kasetsart University, Bangkok 10900, Thailand; 4 Center for Advanced Studied Agriculture and Food, Kasetsart University, Bangkok 10900, Thailand; 5 Department of Product Development, Faculty of Agro-Industry, Kasetsart University, Bangkok 10900, Thailand

**Keywords:** emulsifying capacity and stability, inhibition of *Candida albicans*, protein extracts, ultrasound-assisted extraction, *Wolffia globosa*

## Abstract

*Wolffia globosa* is an interesting alternative plant-based protein source containing up to 40% protein dry weight. Dried duckweed protein extract (PE) was obtained using ultrasound-assisted extraction (UAE) before isoelectric precipitation (pH 3.5) to yield protein concentrate (PC) and protein solution (PS). The PC was hydrolyzed using Alcalase enzyme to obtain protein concentrate hydrolysate (PCH). Among all fractions, PCH exhibited antimicrobial properties by decreasing populations of *Vibrio parahaemolyticus* and *Candida albicans* at 0.43 ± 1.31 log reduction (66.21%) and 3.70 ± 0.11 log reduction (99.98%), respectively. The PE and PS also showed high solubilities at pH 8 of 90.49% and 86.84%, respectively. The PE demonstrated the highest emulsifying capacity (EC) (71.29%) at pH 4, while the highest emulsifying stability (ES) (~98%) was obtained from the PE and PS at pH 6 and pH 2, respectively. The major molecular weights (Mw) of the PE, PC, PCH and PS were observed at 25, 45, 63 and 100 kDa, with a decrease in the Mw of the PCH (<5 kDa). The PCH contained the highest total amino acids, with aspartic acid and glutamic acid being the major components. The results revealed the antimicrobial and functional properties of duckweed protein and hydrolysate for the first time and showed their potential for further development as functional food ingredients.

## 1. Introduction

Animal farming is a crucial contributor to greenhouse gas emission, and the growing world population demands increasing attention to more sustainable consumption of plant-based products. Decreasing consumption of animal products such as meat, fish, milk and eggs, which are key sources of proteins, and increasing consumption of agricultural yields are important goals to sustain food production [1]. Recently, several plant proteins have become commercially available and shown rapid growth in the food industry [2]. The most often-used plant proteins are soy and wheat protein concentrates, but these are categorized among the eight foods that cause major allergies [3]. Therefore, alternative plant protein sources are gaining interest to increase diet diversity.

Duckweed, *Wolffia globosa*, known in Thai as khai-nam or phum, is the world’s smallest flowering plant. It is commonly found in freshwater habitats in Thailand and many countries in Southeast Asia. This plant is a rich source of nutrients, with 16–41% protein, high levels of minerals and vitamins at 3.5–26%, 17–35% carbohydrate content and 3–9% fat based on dry weight. Variations in the plant’s chemical composition depend on the strain and cultivation methods [4]. Duckweed has a simple structure and a more rapid growth rate than terrestrial plants, with 3.5–6.5 days of doubling time [5]. Growth at the industrial scale could offer an interesting source of alternative plant-based protein. However, few scientific validations of the nutritional value, biological and functional properties and commercial feasibility of duckweed have been conducted.

Recently, ultrasound-assisted extraction (UAE) has been widely used to extract many natural products as a proven and effective method to improve the yield of extracted components, increase the extraction rate and decrease the extraction time [6,7]. UAE is a green technique that facilitates plant protein extraction in less time from many plants. Kadam et al. [8] investigated the enhancement of protein extraction yields from the seaweed *Ascophyllum nodusum*, closely related to duckweed, using ultrasound pretreatment. Results showed that UAE at an amplitude of 68.4 µm increased protein recovery from 7.97 ± 0.26% to 43.13 ± 2.21% and 51.80 ± 1.74% to 57.23 ± 2.31% compared to acid and alkaline extraction alone, respectively, while the extraction time decreased from 60 min to 10 min.

Previous studies revealed that protein hydrolysate prepared by enzyme digestion is rich in bioactive peptides. Some peptides show bioactive antioxidant and antihypertensive properties by inhibiting angiotensin-converting enzyme (ACE) and antimicrobial activity, depending on their structure and molecular weight (Mw). Antimicrobial peptides derived from natural sources are important, as they have low toxicity and high specificity. Görgüç et al. [9] reported that soybean, rice bran and chia flour contained bioactive peptides with antimicrobial properties. In addition, proteins are used as essential ingredients in food applications due to their biological activities and functional properties such as the formation of emulsions and foams [10]. The protein isolate obtained from the protein-rich lupin variety AluProt-CGNA^®^ (LPIA) at pH 3 showed high emulsion capacity (%EC) and emulsion stability (%ES) values of 63% and 99%, respectively [11]. LPIA also exhibited potential use as a stabilizer for oil-in-water (O/W) Pickering emulsions, with more beneficial applications in food industries [12].

Most previous studies focused on the genus *Lemna*, with scant information available on the biological and functional properties of *W. globosa*. Therefore, this study extracted proteins from *W. globosa* using UAE by developing different fractions, including protein concentrate and hydrolysate, through enzymatic hydrolysis and investigated their antimicrobial activities and functional properties. The solubilities and emulsion formations of active peptides were characterized. The results obtained can expand and enhance the use of *W. globosa* as a protein-enriched functional ingredient for further applications in food products.

## 2. Materials and Methods

### 2.1. Materials

Duckweed (*Wolffia globosa*) harvested between May and June 2020 was obtained from Advanced GreenFarm Co., Ltd., Nakhon Pathom, Thailand. The duckweed was dried in a tray dryer at 60 °C for 8 h before being ground with a hammer mill and passed through a 1 mm sieve to obtain duckweed powder.

### 2.2. Composition Analyses of Dried W. globosa

Proximate compositions of dried duckweed powder were determined according to the AOAC standard protocol [13]. The protein and fat were analyzed by the Kjeldahl method with a conversion factor of 6.25 and Soxhlet extraction, respectively. The crude fiber, moisture and ash were determined using a Fibertec system, oven drying and a muffle furnace at 550 °C, respectively. The remaining percentage was indicated as the carbohydrate content.

### 2.3. Microorganisms

Thirty-three bacterial strains were used to screen the antimicrobial activities of *W. globosa* protein and peptides. Gram-positive bacteria were represented by *Bacillus cereus* TISTR687, *Bacillus cereus* ATCC1178, *Bacillus subtilis* TISTR008, *Brochothrix thermosphacta* ATCC11509, *Listeria innocua* DMST9011, *Listeria monocytogenes* 101, *Listeria monocytogenes* 108, *Listeria monocytogenes* 310, *Listeria monocytogenes* ATCC19114, *Listeria monocytogenes* Scott A, *Listeria monocytogenes* V7, *Staphylococcus aureus* TISTR1466, *Staphylococcus aureus* ATCC25923, *Streptococcus pyogenes* DMST4369, *Streptococcus pyogenes* DMST4478, *Streptococcus pyogenes* DMST17020, *Streptococcus pyogenes* DMST26758 and *Streptococcus pyogenes* DMST30653. *Escherichia coli* ATCC8739, *Moraxella phenylpyruvicus* DMST17591, *Pseudomonas aeruginosa* TISTR781, *Pseudomonas fluorescens* TISTR358, *Salmonella* Typhimurium TISTR292, *Salmonella* Weltevreden DMST15677, *Vibrio parahaemolyticus* ATCC17802 and *Vibrio parahaemolyticus* BCC24339 represented Gram-negative bacteria. *Pediococcus acidilactici* TISTR051, *Lactobacillus mesenteroids* TISTR541, *Lactobacillus pentosus* TISTR920, *Lactobacillus plantarum* TISTR850 and *Leuconostoc mesenteroids* TISTR053 represented lactic acid bacteria, while *Candida albicans* TISTR5815 and *Saccharomyces cerevisiae* 5343 were the two representatives of yeast.

### 2.4. Preparation of Protein Fractions

**Figure 1:**
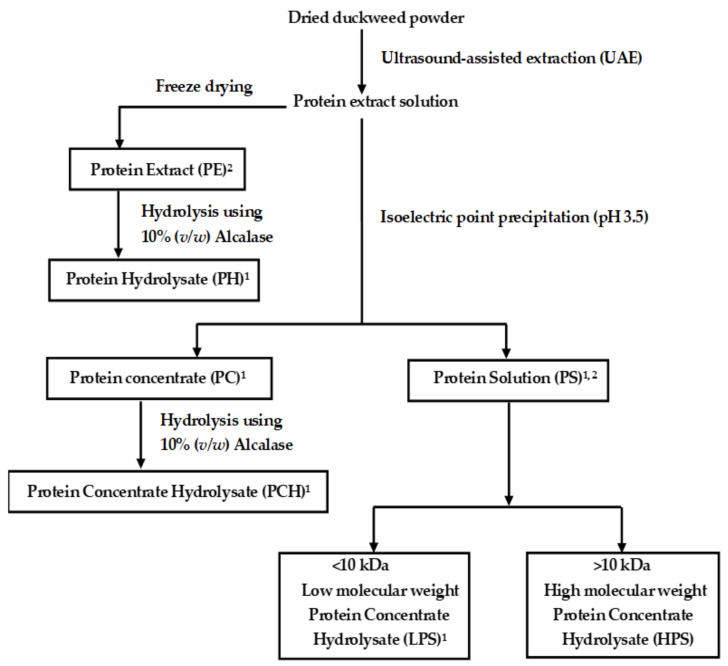
Protein development flow chart. ^1^ Antimicrobial test. ^2^ Functional properties test.

All protein fractions used in this study are summarized in Figure 1. Protein extract (PE) from *W. globosa* was extracted using ultrasound-assisted extraction under the optimized condition, following the method described by Duangjarus et al. [14]. Briefly, dried duckweed powders were mixed with distilled water using a 1:25 ratio of duckweed powder to water and held in the stirred flat-UAE chamber. Ultrasonic frequency at 120 kHz (power level 4) was conducted at room temperature for 15 min, and the pH of the slurry during the process was 6.7. After the ultrasonic treatment process, the slurry was centrifuged at 4 °C and 12,000× *g* for 20 min. The supernatant was evaporated at 60 °C under a vacuum pressure of 72 mbar, freeze-dried and ground as a PE fraction.

To investigate their antimicrobial activities and functional properties, proteins from the PE fractions were precipitated at the isoelectric point (pI) of pH 3.5 to obtain protein concentrate (PC) or precipitate fraction and protein solution (PS) or supernatant fraction. The protein hydrolysate fraction was then developed from the PE and PC through an enzymatic hydrolysis process modified from Silva et al. [15]. Briefly, enzymatic hydrolysis was prepared using Alcalase 2.4 L (enzyme activity ≥ 2.4 activity units/g) at 50 °C for 3 h. The PE and PC at 20% (*w*/*v*) were adjusted to pH 8.0, and then 10% (volume of enzyme/ weight of sample) was added and incubated with slight shaking at 50 °C for 3 h. The enzyme was inactivated by heating at 85 °C for 15 min, followed by cooling in an ice bath until it reached room temperature. The pH of the hydrolyzed mixture was adjusted to 7.0, and it was stored at −20 °C and freeze-dried to obtain protein hydrolysate (PH) and protein concentrate hydrolysate (PCH), respectively. The PS were further fractionated using ultrafiltration with a molecular weight cut-off (MWCO) of 10 kDa (Millipore Corporation, Bedford, MA, USA). They were centrifuged at 5000× *g* for 30 min to separate the samples into >10 kDa (retentate) and <10 kDa (filtrate), stored at −20 °C and freeze-dried.

### 2.5. Antimicrobial Assay

#### 2.5.1. Preparation of Test Microorganisms

The 33 bacterial strains, as representatives of yeast and Gram-negative, Gram-positive and lactic acid bacteria, were used to screen the antimicrobial activities of duckweed protein and peptides. They showed antimicrobial activity against *Vibrio parahaemolyticus*-BCC 24339 and *Candida albicans*-TISTR5815. Thus, these two microbial strains were used for triplicate determination of antimicrobial activity. *V. parahaemolyticus* was grown in Mül ler–Hinton broth (MHB) with 3% NaCl (MHBS) at 37 °C, and *C. albicans* was grown in yeast malt broth (YMB) at 25 °C for 24 h before use.

#### 2.5.2. Antimicrobial Test

The antimicrobial property was determined using the standard macrobroth dilution, slightly modified from the Clinical Laboratory Standards Institute (CLSI) guideline document M7-A1 [16]. Test tubes containing 1 mL of *V. parahaemolyticus* in MHBS and *C. albicans* in YMB at double-strength concentration (cell concentration around 4–5 log CFU/mL) were mixed with 1 mL of each sample (at the final sample concentration of 20 mg/mL in water) to give a final volume of 2 mL. Test tubes containing *V. parahaemolyticus* and *C. albicans* were incubated at 37 °C and 25 °C, respectively, for 24 h. Enumerations of bacteria were examined using the spread plate technique. *V. parahaemolyticus* was grown on tryptic soy agar supplemented with 3% NaCl (TSAS), while *C. albicans* was grown on yeast malt agar (YMA). Each agar was then incubated at a suitable temperature for 24 h before the bacterial colonies were counted. Log reduction (LR) was calculated using Equation (1).

Log reduction = log_10_(N_0_/N) (1)

N_0_ = Colony-forming units of the bacteria in positive control at 24 h

N = Colony-forming units of the bacteria in treatment at 24 h

### 2.6. Determination of Functional Properties

#### 2.6.1. Protein Solubility

Protein solubility profiles of samples at different pH values were determined following the method of Piornos et al. [17]. Dry extracts were dispersed in distilled water (10 mg/mL), and the pH values of the suspensions were adjusted to 2, 4, 6, 8 and 10 using 1 M sodium hydroxide (NaOH) or 1 M hydrochloric acid (HCl). The solutions were stirred at 25 °C for 1 h before centrifuging at 8000× *g* and 4 °C for 10 min. The supernatants were 10-fold diluted in distilled water, and protein contents were determined using the Lowry method [18]. The percentage solubility (%S) of the protein extracts at different pH values was calculated using Equation (2).

Solubility (%S) = (Protein supernatant/Total protein) × 100(2)

#### 2.6.2. Emulsifying Capacity (EC) and Emulsifying Stability (ES)

Determination of EC and ES at different pH values (2, 4, 6, 8 and 10) was performed according to the method described by Piornos et al. [17] with a slight modification. Ten milliliters of protein solution (10 mg/mL) with varying pH values was homogenized with 10 mL of sunflower oil at 10,000 rpm for 30 s. An equal volume (10 mL) of sunflower oil was then added to the mixture before homogenizing at the same speed for 90 s. The tubes were left to stand for 1 h at room temperature. The total height of the liquid sample and emulsion layers was measured at 0 and 24 h. The EC_24_ was the relation of the height of emulsion layers at 24 h (H_EL_) to the total height of the liquid sample (H_T_), whereas the ES was calculated by the ratio of the EC_24_ to the EC at the initial time (EC_0_), following Equations (3) and (4).

EC (%) = (H_EL_/H_T_) × 100 (3)

ES (%) = (EC_24_/EC_0_) × 100 (4)

### 2.7. Characterization of Proteins and Peptides

#### 2.7.1. Molecular Weight (Mw) Determination of Proteins and Peptides

The Mw of proteins and peptides was investigated using sodium dodecyl sulfate–polyacrylamide gel electrophoresis (SDS-PAGE) according to Laemmli [19]. Duckweed protein and peptide solutions (10 µg/µL) were loaded onto 16% polyacrylamide gel and run at 60 V for 5 min before continued running at 100 V for 90 min. The gel was stained using Coomassie blue. TriColor Broad Protein Ladder (3.5–245 kDa) was used to evaluate protein size.

#### 2.7.2. Amino Acid Composition Analysis

Amino acids were determined according to the AOAC method [18], with some modifications. Dried samples were digested with 8 mL of 6 M HCl at 110 °C for 22 h and then cooled before adding 4.8 mL of 10 M NaOH. The volume was increased to 25 mL with distilled water before filtering through filter paper no. 40 and centrifuging at 10,000× *g* for 10 min. Amino acids were analyzed by using an Amino Acid Analyzer (L-8900, Hitachi, Japan). Each sample (1 μL) was injected into an ion exchange column (column size: 4.6 × 60 mm) at 40 °C with detection at 338 nm. The amino acid composition was expressed as mg of amino acids per g of protein.

### 2.8. Statistical Analysis

One-way analysis of variance followed by Duncan’s multiple comparison test was used for datasets with more than two samples. For all analyses, IBM SPSS statistic Version 23.0 (Thaisoftup Co., Ltd., Bangkok, Thailand) with significant difference at the 95% confidence level was used.

## 3. Results

### 3.1. The Compositions of Dried W. globosa

Proximate compositions based on the dry weight of the dried duckweed powder or starting material in this study are shown in Table 1. The results presented show that the main components of dried duckweed powder were proteins (33.16%) and carbohydrates (36.73%), followed by ash 14.58%, which may represent the mineral content. Low fat content (~3%) was also observed.

### 3.2. Antimicrobial Properties

After screening, the antimicrobial activities of each protein and protein hydrolysate fraction on the 33 microbial strains are shown in Supplementary Materials. The five fractions PH, PS, LPS, PC and PCH (at the final sample concentration of 10 mg/mL in water) were selected based on their percentage yields and activities to further investigate the antimicrobial activities of *V. parahaemolyticu*s and *C. albicans* using a macrobroth dilution assay. The results in Table 2 show that the LPS had no inhibition effect on the reduction of *V. parahaemolyticus* and *C. albicans*. By contrast, the PH and PS reduced *V. parahaemolyticus* population by 0.12 ± 0.46 log reduction (30.83%) and 0.21 ± 0.17 log reduction (43.70%), respectively. In addition, the PC reduced *C. albicans* population by 0.31 ± 0.22 log reduction (53.14%), while the PCH inhibited *V. parahaemolyticus* and *C. albicans* by 0.43 ± 1.31 log reduction (66.21%) and 3.70 ± 0.11 log reduction (99.98%), respectively. Therefore, the PCH fraction at the final protein concentration of 5 mg/mL showed the highest efficiency in decreasing *V. Parahaemolyticus* and *C. albicans* populations. The PS partially obtained from the development process did not show effective antimicrobial activities. However, a high protein content (37%) was observed in this fraction, similar to the PE (41%). Therefore, the functional solubility and emulsifying properties, which are important properties of proteins and useful for further food applications of the PE and PS, were evaluated to take full advantage of all the valuable fractions separated by this fractionation procedure.

### 3.3. Functional Properties of Duckweed Protein

#### 3.3.1. Effect of pH on Protein Solubility

**Figure 2:**
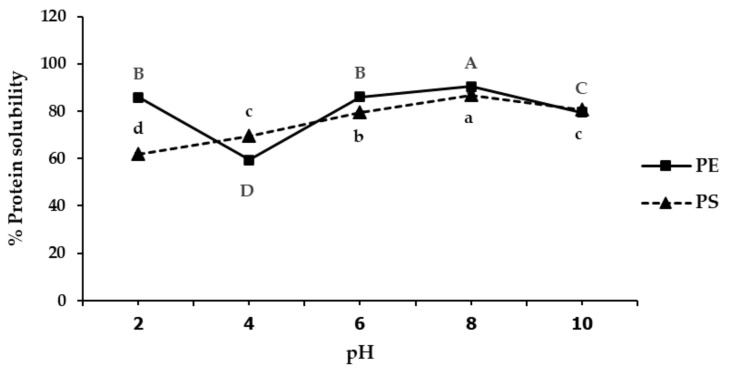
Effect of pH on solubility of protein extract (PE) and protein solution (PS). Mean ± standard deviations from triplicate analyses of PE and PS with different capital and small letters, respectively, are significantly different (*p* < 0.05) within samples.

The effect of pH on protein solubility was investigated in PE and PS. Protein solubility patterns for both fractions were different, as shown in Figure 2. The lowest PE solubilities were found at pH 4 (59.40%), while the highest solubilities were observed at pH 8 (90.49%). Furthermore, PS showed the lowest and highest solubilities at pH 2 (61.82%) and pH 8 (86.84%).

#### 3.3.2. Effect of pH on Emulsifying Property

**Figure 3:**
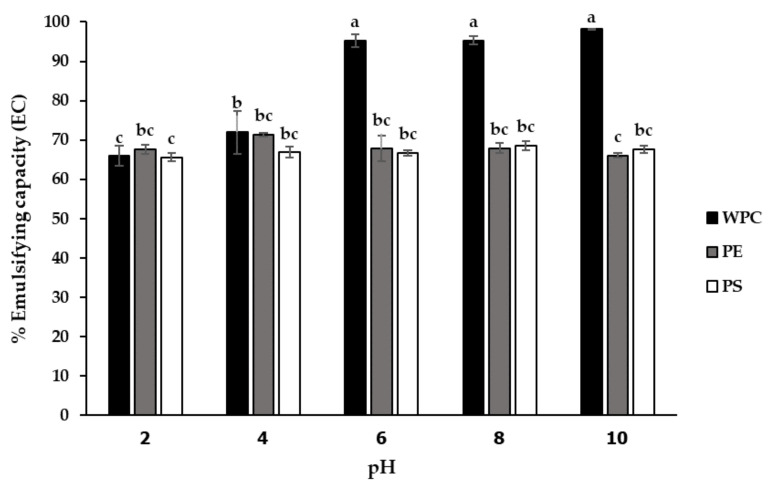
Effect of pH on emulsifying capacity (EC) of protein extract (PE) and protein solution (PS) compared to whey protein concentrate (WPC). Mean ± standard deviations from triplicate analyses of all samples with different small letters are significantly different (*p* < 0.05).

To explore the possible applications of the PE and PS as functional ingredients, the emulsifying capacity (EC) and emulsifying stability (ES) of these protein fractions were compared to whey protein concentrate (WPC), which is a common emulsifier used in many commercial food applications. Figure 3 shows the EC of the WPC, PE and PS at five different pH values (range 2–10). No significant difference was found in %EC between the PE and PS, but both fractions exhibited lower %EC than the WPC due to their lower protein content. When comparing the PE and PS, the highest EC was observed in the PE fraction at pH 4 (71.29%), indicating the optimal emulsifying capacity of this duckweed protein extract. The WPC presented the highest EC at pH 10 (98.15%), while the PS presented a similar EC profile pattern to the WPC, with its highest EC (68.52%) at pH 8 as its highest solubility condition. By contrast, the PE showed the highest EC (71.29%) at pH 4, which was its lowest solubility condition.

**Figure 4:**
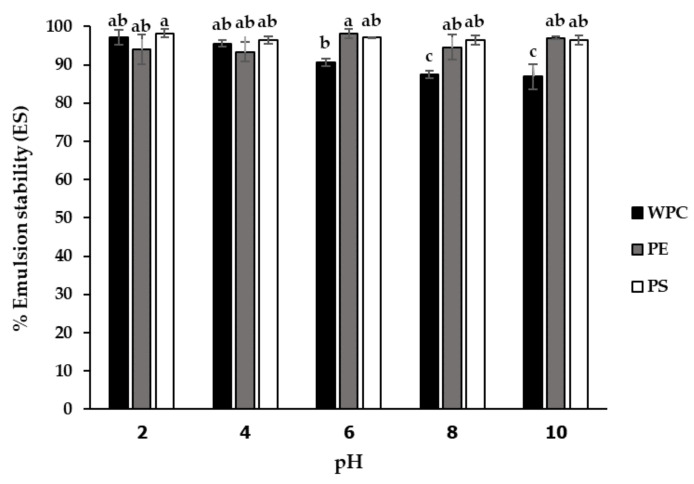
Effect of pH on emulsifying stability (ES) of protein extract (PE) and protein solution (PS) compared to whey protein concentrate (WPC). Mean ± standard deviations from triplicate analyses of all samples with different small letters are significantly different (*p* < 0.05).

Moreover, the emulsifying stability (ES) was evaluated by observing the emulsion layer at 24 h after emulsion preparation. The results showed that emulsions prepared with the PE, PS and WPC yielded good ES stability at above 85% for all evaluated samples. Figure 4 shows no significant differences in ES of the PE and PS for all pH values. The PE and PS recorded the highest ES of 98% at pH 6 and pH 2, respectively with improved emulsifying stability compared to the commercial emulsifier WPC.

### 3.4. Characterization of Duckweed Proteins and Peptides

#### 3.4.1. Determination of Molecular Weight (Mw)

**Figure 5:**
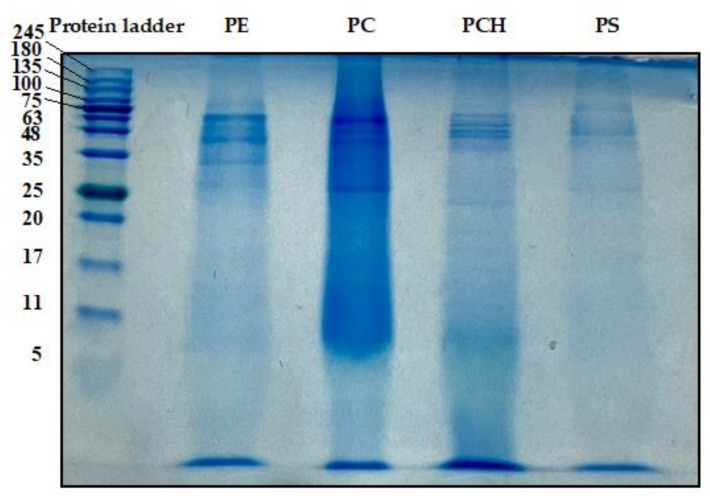
Molecular weight profiles of protein extract (PE), protein concentrate (PC), protein concentrate hydrolysate (PCH) and protein solution (PS) analyzed by SDS-PAGE.

To investigate the Mw of each active duckweed protein fraction, the samples were analyzed by SDS-PAGE (Figure 5). The results showed major bands of the PE at 25, 30, 45, 63 and 100 kDa. PC obtained from protein precipitation showed stronger bands at 25, 45, 50 and 63 kDa. Relative to the PC, the PCH exhibited weaker bands at the same Mw, but a darker lane of low Mw (<5 kDa) was observed compared to the protein ladder. Moreover, the PS fraction also showed weak bands at 25 and between 48–63 and 100 kDa, indicating the lower protein content of this fraction after precipitation of the PC from the PE. 

#### 3.4.2. Amino Acid Analysis

To determine the potential use of the PCH as an antimicrobial, as well as the PE and PS for functional food ingredients to improve emulsion, the amino acid compositions of these samples were analyzed, with the results presented in Table 3. Comparison of amino acid compositions between the protein fractions (PCH and PS) and the PE showed that the total amino acids of the PCH increased, whereas a decrease in total amino acids was observed in the PS. This tendency was in accordance with the higher protein content in the PCH. The major amino acids in the PE were aspartic acid, glutamic acid and alanine at 37.2, 34.8 and 16.2 mg/g sample, respectively, while the PCH contained high aspartic acid, glutamic acid and leucine, presenting at 22.9, 28.9 and 19.6 mg/g sample, respectively. In addition, the PS showed high content of aspartic acid, glutamic acid and alanine as the major amino acids at 24.58, 21.66 and 10.65 mg/g sample, respectively. The PCH fraction presented the highest total amino acid profiles (203.76 mg/g) compared to the PE and PS.

We also compared the amino acid compositions (% based on total amino acid) of the PE, PCH and PS with various plant protein isolates including microalgae, soy, pea, lupin and oat from the review of Gorissen et al. [20] (Table 4). We observed that aspartic acid was the most abundant amino acid in the PE and PS, while the PCH was glutamic acid-rich, which was similar to other plant proteins. The total amino acids of all duckweed proteins from *W. globosa* were comparable to the others, but the percentages of alanine and valine of the PE, PCH and PS were higher than those of soy, pea, lupin and oat. Valine is an essential amino acid that is important for optimal growth in infants and children, and it improves the nitrogen balance in adults, leading to wide applications in the pharmaceutical and food industries [21]. Alanine is an important amino acid involved with sugar metabolism and maintaining the body’s blood sugar balance [22]. These three duckweed proteins from *W. globosa* were rich in histidine, which is an essential amino acid associated with several health benefits including inflammatory, glucoregulatory, antioxidant and body weight management [23]. A combination of different plant-based proteins may be an alternative choice to achieve s higher-quality protein blend, utilizing the large variability of amino acid compositions among plant-based protein sources [20].

## 4. Discussion

The antimicrobial activities of the PCH exhibited the highest log reduction for *V. parahaemolyticus* and *C. albicans*, which are foodborne pathogenic microorganisms. This might relate to the high protein content of the PCH (~60%) compared to the other fractions (protein content ~20–40%). The protein hydrolysate fractions (PH and PCH) derived from enzymatic hydrolysis also displayed increased antimicrobial activities compared to the PE (data not shown) and PC, respectively. However, fractionation of protein solutions with lower Mw values did not affect the inhibition of microbial populations when comparing the PS and LPS. Therefore, further investigation is required to determine the antimicrobial activities of different fractions of protein and peptide hydrolysates from *W. globosa*.

According to the results regarding the PE solubilities, the lowest solubility of proteins relates to a pI value where the net charge of the molecules is zero and repulsive forces between protein molecules are reduced, promoting their aggregation and precipitation. At pH values lower and higher than this pI, proteins carry positive and negative net charges, respectively, and exhibit higher solubility [11]. This solubility behavior was also reported for proteins derived from other legumes such as chickpeas [24], lentils [25] and lupins [26]. The different protein precipitations of the PS and PE might be due to the lower protein and higher carbohydrate content of the PS. Carbohydrates in the PS inhibited precipitation of proteins at the isoelectric point (pI). Burgos-Diaz et al. [11] reported that the positive charge of proteins interacted with the negative charge of polysaccharides to form a stable electrostatic complex when the pH decreased below the pI of proteins, resulting in improvement of protein solubility. The oligosaccharide chain of polysaccharides also contributed to the solubility of proteins [27].

The PE and PS demonstrated a good effect on emulsifying capacity and stability. Lestari et al. [28] suggested that EC strongly related to the amount of soluble protein as protein unfolding occurred and exposed the most lipophilic functional groups at extremely alkaline or acidic pH values. A more soluble protein produces rapid migration to the oil–water interface, hence favoring the formation and stability of emulsions. Several studies have shown a relationship between EC and protein solubility in beach peas, breadfruit and chickpeas [24,29,30]. Although the PE showed the highest EC value at its lowest solubility condition, this behavior concurred with Dee et al. [31], who reported that a protein unfolding at its pI exposed more sites for protein-surface contact, resulting in greater surface activity such as emulsifying and foaming properties. Furthermore, emulsions can be stabilized through interactions between proteins and carbohydrates that reduce surface tension between oil and aqueous phases, thereby augmenting viscosity [32], leading to good ES stability at above 85% for all evaluated samples. The higher carbohydrate contents of the PE and PS improved the association between proteins and polysaccharides. This combination, under appropriate conditions, formed multi-layered membranes, resulting in increased emulsion stability [33]. The association of some proteins resulted in the assembly of amino acids that exposed more hydrophobic groups. Greater interactions with the lipid phase were attributed to the higher ES values of the PE and PS [34]. The EC and ES of the PE and PS were comparable to the protein isolate from the lupin with the highest EC (63%) and ES (99%) at pH 3 reported by Burgos-Diaz et al. [11]. The PE and PS both showed good functionality with high solubility and emulsifying properties, as well as potential for further use as ingredients in functional foods.

The Mw of *W. globosa* protein decreased after enzymatic hydrolysis, similar to the peptide CcDef3, extracted from *Capsicum chinense* fruit, which had a low Mw at 6.5 kDa. This fraction exhibited high inhibition activity on the growth of yeast *C. albicans* (86%), followed by *C. buinensis* (69%) and *C. tropicalis* (21%). Moreover, Nasir et al. [35] found a low Mw of shortfin scad muscle protein hydrolysate (SSMPH), with high potential bioactivity and functional properties particular to SSMPH peptides (<3 kDa) that exhibited the highest ACE inhibitory activity and DPPH scavenging activity. In addition, Nieuwland et al. [36] reported that high concentrations of glutamic acid, arginine and glycine are conditionally essential amino acids in the human diet. Therefore, consumption of the PE and PCH could assist people who suffer from limited synthesis of these amino acids due to pathophysiological conditions. Several plant-based proteins are low in lysine and leucine, but not the *W. globosa* proteins. Therefore, *W. globosa* shows promise as an interesting protein source to develop food products with appropriate amino acid compositions. Additionally, the highest antimicrobial activity of the PCH might also relate to its lower Mw (rich in <5 kDa) and higher amino acid content (203.76 mg/g) compared to the other fractions. High contents of aspartic acid and glutamic acid as residues of anionic peptides were also observed in the PCH. Both amino acids facilitate the binding of metal ions, which is necessary for pathogen survival and virulence, leading to resistance of microbial growth [37].

## 5. Conclusions

This study demonstrated the antimicrobial and functional properties of protein extract from duckweed *W. globosa* and its fractions. The results demonstrated that the PCH inhibited the growth of *C. albicans* and decreased the growth of *V. parahaemolyticus*. For functional properties, the PE and PS showed good solubility, with the highest protein solubility at pH 8. The PE presented a higher EC at pH 4 than the PS, but it was still lower than that of the WPC at pH 2. The PE and PS showed higher ES than the WPC in all evaluated pH conditions. Major Mw values of 25, 45, 63 and 100 kDa were observed in all samples, with the lowest Mw being that of the PCH (less than 5 kDa). Relative to the PE, total amino acids in the PCH increased, whereas the PS contained lower totals of amino acids. The major amino acids in these three fractions were aspartic acid and glutamic acid. Thus, the valuable fractions obtained from this extraction and fractionation process of proteins and peptides from duckweed *W. globosa* using green UAE technology can expand the utilization of duckweed as a high-value functional food ingredient. Further research is necessary to develop the fractionation process of the PCH to increase antimicrobial activity efficiency. An improved understanding of the compositions and mechanisms involved is required to elucidate the effects of *W. globosa* protein and peptide extracts on functional properties.

## Figures and Tables

**Table 1:** Proximate compositions of dried *W. globosa*.

Components	Dried *W. globosa* Powder (% Dry Basis)
Moisture	8.61 ± 0.02
Protein	33.16 ± 1.91
Carbohydrate	36.73 ± 2.03
Ash	14.58 ± 0.10
Crude fiber	12.49 ± 0.17
Fat	3.03 ± 0.19

**Table 2:** Log reduction of *V. parahaemolyticus* and *C. albicans* after 24 h incubation with different *W. globosa* fractions: protein hydrolysate (PH), protein concentrate (PC), protein concentrate hydrolysate (PCH), protein solution (PS) and low molecular weight protein solution (LPS).

Sample	Log Reduction	
	*V. parahaemolyticus*	*C. albicans*
PH	0.12 ± 0.46 ^ns^	NI
PC	NI	0.31 ± 0.22 ^b^
PCH	0.43 ± 1.31 ^ns^	3.70 ± 0.11 ^a^
PS	0.21 ± 0.17 ^ns^	NI
LPS	NI	NI

NI; No inhibition effect. Mean ± standard deviations from triplicate analyses in a column with different small letters are significantly different (*p* < 0.05). ^ns^ No significant difference (*p* > 0.05).

**Table 3:** Amino acid compositions of protein extract (PE), protein concentrate hydro-lysate (PCH) and protein solution (PS).

Amino Acid	Amount (mg/g)		
	PE	PCH	PS
Lys	6.58	11.04	5.50
His	8.79	12.76	8.83
Arg	6.57	12.40	7.07
Asp	37.20	22.85	24.58
Glu	34.80	28.89	21.67
Ser	6.57	9.18	3.98
Thr	5.70	8.61	3.12
Ala	16.21	15.57	10.65
Val	14.94	16.90	9.51
Ile	7.86	9.60	4.65
Leu	15.19	19.56	8.46
Met	0.33	0.15	0.21
Phe	8.47	11.21	5.05
Tyr	1.67	5.58	2.35
Cys	0.00	0.00	0.00
Gly	11.92	12.28	7.11
Pro	7.04	7.19	2.89
Total amino acids	189.84	203.77	125.63

**Table 4:** Amino acid profiles as a percentage of total amino acids of duckweed proteins from *W. globosa* compared to various plant-based proteins.

Amino Acid	Percentage							
	PE	PCH	PS	Micro-Algae	Soy	Pea	Lupin	Oat
Lys	3.66	5.37	4.72	9.68	6.59	8.13	5.08	3.40
His	4.70	6.34	7.08	1.88	2.91	2.77	2.91	2.36
Arg	3.66	5.85	5.50	9.14	9.30	10.21	13.32	8.12
Asp	19.34	11.22	19.65	NA	NA	NA	NA	NA
Glu	18.30	14.15	17.30	15.32	24.03	22.32	30.02	28.80
Ser	3.66	4.83	3.14	5.65	6.59	6.23	6.05	5.76
Thr	3.14	4.39	2.36	5.65	4.46	4.33	3.87	3.93
Ala	8.36	7.80	8.65	10.75	5.43	5.54	4.12	5.76
Val	7.84	8.29	7.86	5.65	4.26	4.67	3.39	5.24
Ile	4.18	4.88	3.93	3.23	3.68	3.98	3.63	3.40
Leu	7.84	9.27	6.29	10.75	9.69	9.86	7.75	9.95
Met	0.16	0.05	0.16	0.00	0.58	0.52	0.48	0.26
Phe	4.18	5.37	3.93	5.65	6.20	6.40	4.36	7.07
Tyr	1.05	2.93	1.57	3.23	4.26	4.50	4.60	3.93
Cys	0.00	0.00	0.00	0.27	0.39	0.35	0.48	1.05
Gly	6.27	5.85	5.50	6.99	5.23	4.84	5.08	4.45
Pro	3.66	3.41	2.36	6.18	6.40	5.36	4.84	6.54

NA; Not Analysis.

## Data Availability

The datasets generated for this research are available on request to the corresponding author.

## Supplementary Materials

The following supporting information can be downloaded at https://www.mdpi.com/article/10.3390/foods11152348/s1: Table S1: Log reduction of 33 representative microbial strains tested for 24 h with PE at different concentrations and solvents; Table S2: Log reduction of 16 representative strains tested for 24 h with protein extract (PE) and protein hydrolysates including protein hydrolysate using Alcalase (PH), low Mw of protein hydrolysate using Alcalase (LPH), high Mw of protein hydrolysate using Alcalase (HPH), protein hydrolysate using Viscozyme (PHV) and low Mw of protein hydrolysate using Viscozyme (LPHV).
