# Supplementary material for: Antimicrobial and Functional Properties of Duckweed (Wolffia globosa) Protein and Peptide Extracts Prepared by Ultrasound-Assisted Extraction
Source: Foods. 2022 Aug 5;11(15):2348. doi: 10.3390/foods11152348 (PMC9367961; doi:10.3390/foods11152348)
Supplement: Supplementary file 1 [file foods-11-02348-s001.zip › foods-1831297-supplementary.pdf]

Supplementary materials

To study the antimicrobial activity of *W. globosa* protein extract (PE), 33 microbial strains as representatives of yeast, gram-positive, gram-negative and lactic acid bacteria were screened for the antimicrobial property using the standard macrobroth dilution same as the assay described in the manuscript. Results in Table S1 indicated that PE dissolved in water at the final sample concentration of 1 mg/mL showed no inhibitory effect against all microbial strains, except for *B. thermosphacta* 11509, *L. plantarum* 850 and *C. albicans* 5815 where growth was reduced by 0.05, 0.19 and 0.25 log reduction, respectively. The final sample concentration was increased to 5 mg/mL by dissolving in 5% DMSO. At higher concentrations, PE slightly increased the inhibition of microbial strains, except for *L. Plantarum* and *C. albicans*.

Table S1. Log reduction of 33 representative microbial strains tested for 24 h with PE at different concentrations and solvents.

Bacterial strain	Log reduction	
	1 mg/mL of water	5 mg/mL of 5% DMSO
Gram-positive bacteria		
B. cereus 687	NI	0.04
B. cereus 1178	NI	0.05
B. subtilis 008	NI	NI
B. thermosphacta 11509	0.05	0.12
S. aureus 1466	NI	0.22
S. aureus 25923	NI	NI
L. monocytogenes 101	NI	NI
L. monocytogenes 108	NI	NI
L. monocytogenes 310	NI	NI
L. monocytogenes Scott A	NI	0.1
L. monocytogenes V7	NI	0.27
L. monocytogenes 19114	NI	0.26
L. innocua 9011	NI	0.12
S. pyogenes 17020	NI	0.03
S. pyogenes 26758	NI	NI
S. pyogenes 30653	NI	0.92
S. pyogenes 4369	NI	NI
S. pyogenes 4478	NI	NI
Gram-negative bacteria		
E. coli	NI	NI
S. Typhimurium 292	NI	0.44
S. Weltevreden 15677	NI	NI
V. parahaemolyticus 24339	NI	0.07
V. parahaemolyticus 17802	NI	NI
P. aeruginosa 781	NI	NI
P. fluorescens 358	NI	NI
M. phenylpyruvicus 17591	NI	NI
Lactic acid bacteria		
P. acidilactici 051	NI	NI
L. mesenteroids 053	NI	NI
L. mesenteroids 541	NI	0.6
L. plantarum 850	0.19	NI
L. pentosus 920	NI	NI
Yeast		
C. albicans 5815	0.25	NI
S. cerevisiae 5343	NI	NI

NI; No inhibition effect

Sixteen microbial strains including *B. cereus, B. thermosphacta, S. aureus, L. monocytogenes, L. monocytogenes, L. innocua, Strep. pyogenes, E. coli, S.* Typhimurium, *S.* Weltevreden, *V. parahaemolyticus, P. aeruginosa, P. fluorescens, M. phenylpyruvicus, P. acidilactici, L. mesenteroids, L. plantarum, L. pentosus, C. albicans* and *S. cerevisiae* were selected to investigate the antimicrobial effects of different protein fractions obtained following the enzymatic hydrolysis process and centrifugal ultrafiltration. To compare the enzyme efficiency, PE was hydrolysed by 10% (v/w) Alcalase and 10% (v/w) Viscozyme for 3 h under the optimal pH and temperature of each enzyme. The protein hydrolysates were separated by a 10 kDa molecular weight cut-off (MWCO) filter to obtain two fractions of *W. globosa* protein hydrolysates: larger than 10 kDa (retentate) and smaller than 10 kDa (filtrate). Results of PE and five protein hydrolysates derived from the enzymatic hydrolysis and filtration at the final sample concentration of 5 mg/mL in water are shown in Table S2. Protein hydrolysate using Alcalase (PH), low Mw of protein hydrolysate using Alcalase (LPH) and high Mw of protein hydrolysate using Alcalase (HPH) showed a promising tendency as antimicrobial peptides by 1 log reduction (>90%) on *C. albicans, L. plantarum* and *P. aeruginosa,* whereas protein hydrolysate using Viscozyme (PHV) and low Mw of protein hydrolysate using Viscozyme (LPHV) showed lower efficiency to reduce the microbial populations. These results suggested that Alcalase has more specificity to protein than Viscozyme, leading to increased bioactive peptide release. After considering the screening results associated with the percentage yield of each fraction, we selected five fractions consisting of PH, PC, PCH, PS and LPS to determine antimicrobial activity against *V. parahaemolyticus* and *C. albicans* using broth dilution assay.

Table S2. Log reduction of 16 representative strains tested for 24 h with protein extract (PE) and protein hydrolysates including protein hydrolysate using Alcalase (PH), low Mw of protein hydrolysate using Alcalase (LPH), high Mw of protein hydrolysate using Alcalase (HPH), protein hydrolysate using Viscozyme (PHV) and low Mw of protein hydrolysate using Viscozyme (LPHV).

**Bacterial strain**	**Log reduction**					
	**PE**	**PH**	**LPH**	**HPH**	**PHV**	**LPHV**
Gram-positive bacteria						
B. cereus 687	NI	NI	0.18	NI	NI	NI
B. thermosphacta 11509	NI	NI	NI	NI	NI	NI
S. aureus 25923	NI	NI	NI	0.12	NI	NI
L. monocytogenes 108	0.11	0.09	NI	NI	0.27	NI
L. monocytogenes V7	0.14	0.18	NI	NI	NI	NI
L. monocytogenes 19114	NI	NI	0.15	0.33	0.13	NI
L. innocua 9011	NI	NI	NI	NI	NI	NI
Gram-negative bacteria						
S. Typhimurium 292	0.05	0.04	NI	NI	NI	NI
S. Weltevreden 15677	0.13	0.03	NI	NI	NI	NI
V. parahaemolyticus 24339	NI	0.94	NI	0.51	0.09	0.45
P. aeruginosa 781	0.14	0.37	1.2	1.01	0.37	NI
P. fluorescens 358	0.24	0.44	NI	NI	0.44	NI
Lactic acid bacteria						
L. mesenteroids 541	NI	NI	NI	NI	0.11	NI
L. plantarum 850	0.67	0.45	1.41	NI	0.7	NI
Yeast						
C. albicans 5815	0.76	1.51	NI	0.33	0.3	NI
S. cerevisiae 5343	NI	NI	0.88	0.91	NI	NI

NI; No inhibition effect
